# Editorial: The Border Between Kitavirids and Nege-Like Viruses: Tracking the Evolutionary Pace of Plant- and Arthropod-Infecting Viruses

**DOI:** 10.3389/fpls.2022.932523

**Published:** 2022-05-24

**Authors:** Pedro Luis Ramos-González, Hideki Kondo, Sergey Morozov, Nikolaos Vasilakis, Arvind Varsani, Mengji Cao, Juliana Freitas-Astúa

**Affiliations:** ^1^Applied Molecular Biology Laboratory, Biological Institute of São Paulo, São Paulo, Brazil; ^2^Institute of Plant Science and Resources, Okayama University, Kurashiki, Japan; ^3^A. N. Belozersky Institute of Physico-Chemical Biology, Lomonosov Moscow State University, Moscow, Russia; ^4^Institute for Human Infections and Immunity, University of Texas Medical Branch at Galveston, Galveston, TX, United States; ^5^Center for Fundamental and Applied Microbiomics, The Biodesign Institute, Arizona State University, Tempe, AZ, United States; ^6^Citrus Research Institute, Chinese Academy of Agricultural Sciences, Beibei, China; ^7^Laboratório de Fitopatologia, Embrapa Mandioca e Fruticultura, Cruz das Almas, Brazil

**Keywords:** *Kitaviridae*, *Cilevirus*, *Higrevirus*, negeviruses, *Blunervirus*, *Brevipalpus*

Since the disclosure of the citrus leprosis virus C (CiLV-C) genome and the creation of the family *Kitaviridae*, viruses of the eight accepted species within three genera of kitavirids have become intriguing pieces of the plant-infecting virome. With genomes split into two, three, or four single-stranded (+) RNA molecules, most of the kitaviruses produce non-systemic infections that, in the case of citrus leprosis disease, entail serious economic losses to the citrus crop. Phylogenetic analyses based on the RNA-dependent RNA polymerase proteins indicate that kitavirus closest relatives are a group of unclassified arthropod-infecting viruses tentatively known as negeviruses and nege/kita-like viruses.

Contributions gathered in this Research Topic offer data that broaden the boundaries of the diversity of kitaviruses and nege/kita-like viruses, provide, with a special emphasis on the cilevirus CiLV-C, new insight into the interaction between kitavirids and plants, and increase the understanding of the role of some viral open reading frames (ORF) typical to these viruses. They also demonstrate the vectorial activity of mites of the genus *Brevipalpus* for some of the newly described kitaviruses and postulate a hypothesis describing the movement of CiLV-C virions in mites. Particular attention to the forces modulating the population structure of CiLV-C, and the likely evolutionary pathways that could give rise to the diversity of viruses in the family *Kitaviridae* are also addressed.

Five new kitaviruses are reported. The description of three strains of passion fruit green spot virus representing a typical member of the genus *Cilevirus* revealed, however, that their RNA2 molecules have highly variable 5′-ends, with putative orphan ORFs exclusively found in some of these isolates (Ramos-González, dos Santos, et al.). Hibiscus yellow blotch virus, recently accepted as a cilevirus, has a chimeric genome with an RNA1 similar to that of hibiscus green spot virus (HYBV, genus *Higrevirus*), and its RNA2 displays a novel organization in the family, truncated at its 5′-end but incorporating an extra ORF at its 3′-end (Olmedo-Velarde et al.). The remaining three kitavirids were found infecting ornamental plants and are indicated as tentative cileviruses, but their genomes also show divergences from the recognized members of the genus. The genomic organization of Solanum violifolium ringspot virus, Ligustrum chlorotic spot virus, and Ligustrum leprosis virus resembles that in cileviruses, excluding the absence of the 5′-end region of the RNA2, upstream of the ORF *p61*, reminiscent of the HYBV RNA2 (Ramos-González, Chabi-Jesus, et al.). Studies on virion morphology, transmission by *Brevipalpus* mites, and morphopathology of the infected plant cells complement the molecular characterization of these viruses and improve the integral understanding of the family *Kitaviridae*.

In parallel, a meta-transcriptomic approach to study the virome of 15 aphids populations infesting barley fields in combination with searches on the transcriptome shotgun assembly (TSA) libraries of several arthropods and plants reveal viruses of a dozen of RNA families, including some novel nege/kita-like viruses (Kondo et al.). Barley aphid RNA viruses 1–4 (BARV 1–4) have non-segmented genomes comprising a large ORF (RdRp) followed by two putative structural protein genes for a predicted glycoprotein and the membrane protein SP24, also present in kitavirids. BARV 1–4 cluster with other nege/kita-like viruses forming new genera tentatively designated as Centivirus and Aphiglyvirus. Phylogenetically, they contribute to filling the gaps between the lineages of negeviruses and kitaviruses.

The interplay of plant-kitavirids is addressed in two studies. The global plant response to the infestation by viruliferous *Brevipalpus* mites shows that the CiLV-C infection triggers a progressive reprogramming of the plant transcriptome; namely the activation of the SA-mediated pathway, including ROS burst and HR, and the downregulation of the JA/ET-mediated pathways (Arena et al.). The transient expression of the CiLV-C P61 protein mimics the responses typically observed during CiLV-C-plant interaction indicating that P61 is a putative viral effector causing the HR-like symptoms associated with the infection, likely the outcome of an incompatible interaction. Furthermore, using several strategies to drive the ectopic expression of CiLV-C ORFs, the RNA silencing suppressor (RSS) activity of the viral proteins is investigated (Leastro et al.). Although local RSS activities were not found, the expression of P29 and P15 enhances the pathogenicity of potato virus X (PVX) resulting in the death of the infected tobacco plants. Meanwhile, PVX-p61 infection resulted in HR, corroborating the evidence that the expression of this protein activates plant defense mechanisms.

CiLV-C population study presented in this Research Topic includes the largest collection of samples from leprosis-affected citrus trees ever considered, including herbarium samples collected since the early twentieth century (Chabi-Jesus et al.). Eighteen complete or near-complete viral genomes were recovered, and the examination of the dataset suggests that the CiLV-C population consists of the major lineages CRD and SJP, unevenly distributed in citrus orchards, plus a third one called ASU, represented by a single isolate found in an herbarium sample collected in Asuncion, Paraguay, in 1937. Viruses from these three lineages show signs of inter-clade recombination events. The viral origin by recombination of the 5′-end of the RNA2 in cileviruses and their putative acquisition from a heterologous source is also suggested in a study based on the prediction and computational analyses of 3D protein structures of P15 proteins. These small and poorly conserved proteins are encoded by ORFs unique to cileviruses (Ramos-González, Pons, et al.). Beyond cileviruses, recombination seems to be a common feature in the evolution of kitaviral genomes. The recombinant evolutionary origin of kitaviral genomes is emphasized in a thorough study that includes kitaviruses, nege/kita-like viruses, and also virus-like RNA assemblies found by searching TSA, as the quadripartite blunervirus-like RNA detected in *Paulownia tomentosa* (Morozov et al.).

Finally, the Research Topic presents a paper compiling what is known about cilevirus transmission by *Brevipalpus* mites (Tassi et al.). It describes the presence of CiLV-C particles aligned in the intercellular spaces between adjacent cells at the caeca and the podocephalic gland of mites, which supports the hypothesis of the virion paracellular movement.

## Author Contributions

All authors listed have made a substantial, direct, and intellectual contribution to the work and approved it for publication.

## Funding

The authors were grateful to CAPES, CNPq, and FAPESP for scholarships, research fellowships, and research grants associated with this work (CAPES PNPD20132154-33141010001P4-PNPD-IBSP, 88882.157041/2017-01, CNPq 312528/2020-5, FAPESP 2017/50222-0, 2017/50334-3, and 2019/25078-9).

## Conflict of Interest

The authors declare that the research was conducted in the absence of any commercial or financial relationships that could be construed as a potential conflict of interest.

## Publisher's Note

All claims expressed in this article are solely those of the authors and do not necessarily represent those of their affiliated organizations, or those of the publisher, the editors and the reviewers. Any product that may be evaluated in this article, or claim that may be made by its manufacturer, is not guaranteed or endorsed by the publisher.

